# Health Tract, No. 138

**Published:** 1863-05

**Authors:** 


### HEALTH TRACT, No. 138.

### SPECIFICS

ARE such drugs as very certainly cure the ailments or effect the objects for which they are administered. No medicine can be always successful, for man was born to die; but there are some which so uniformly accomplish the end intended that they are very implicitly relied upon. There are specifics moral as well as medicinal, and it may answer a useful purpose to give examples of both.

The best specific for a horse-thief is a hempen halter; never since the world began, has it ever been found necessary to repeat the dose.

If you want to get rid of a troublesome and unprincipled acquaintance, without offending him, lend him five dollars.

A specific for all earthly troubles, not excepting that greater than all of them, a partnership with a virago and a shrew, said to have been the lot of one of the wisest of men, Socrates the husband of Xantippe, as also one of the best of men, the good John Wesley, is a dose of strychnine; but this is jumping out the frying-pan into the fire, for the suicide, the last act of whose life is the deliberate violation of one of the plainest of all the commands of the great and good Father of all, "Thou shalt not kill," must wake up in the life beyond, with that "fearful looking for of judgment," which is the lot of all the wicked.

But there is another kind of specific, wholly different from all these, and of another meaning, it is that of specific directions, medically speaking, for the want of which many a prescription has proved inefficient, and many a valuable life has been lost. A physician once advised a sufferer to apply a mustard plaster to the chest. The next morning the patient returned, worse than before. On more specific inquiry it was ascertained that with becoming faith, particularity and earnestness, the plaster aforesaid had been applied to the chest, but it was to the wooden one at home, which held all the patient's clothing. The doctor's directions were not specific enough. I have often found it very satisfactory as to results, when giving instructions to patients as to that all-important agent in the cure of disease, diet, to put in print the exact items of food to be placed on the table, adding thereto: "Nothing else." This is specific, clear, sharply defined. Not so the judge in the following case, as no doubt the unfortunate jurors felt to their sorrow:

"If the jury believe, from the evidence, that the plaintiff and defendant were partners in the grocery, and that the plaintiff bought out the defendant, and gave his note for the interest, and the defendant paid for the note by delivering to the plaintiff a cow, which he warranted not breachy, and the warranty was broken by reason of the breachiness of the cow, and he drove the cow back and tendered her to the defendant, but the defendant refused to receive her, and the plaintiff took her home again, and put a heavy yoke or poke upon her to prevent her from jumping the fence, and by reason of the yoke or poke she broke her neck and died; and if the jury further believe that the defendant's interest in the grocery was worth any thing, the plaintiff's note was worthless and the cow good for nothing, either for milk or beef, then the jury must find out themselves how they will decide the case; for the court, if she understands herself, and she thinks she do, is at a considerable non-plus how such a case should be exactly decided."

